# Comparison between 2D and 3D MEDIC for human cervical spinal cord MRI at 3T

**DOI:** 10.1002/jmrs.433

**Published:** 2020-09-15

**Authors:** Abdullah Asiri, Franky Dimpudus, Nicole Atcheson, Aiman Al‐Najjar, Katie McMahon, Nyoman D. Kurniawan

**Affiliations:** ^1^ Centre for Advanced Imaging University of Queensland Brisbane Australia; ^2^ Radiological Sciences Department College of Applied Medical Sciences Najran University Najran Saudi Arabia; ^3^ Rumah Sakit Premier Surabaya ‐ Ramsay Sime Darby Healthcare Surabaya Indonesia; ^4^ Herston Imaging Research Facility School of Clinical Sciences Institute of Health and Biomedical Innovation Queensland University of Technology Brisbane Australia

**Keywords:** cervical spinal cord, 3 Tesla, MEDIC, grey matter, white matter

## Abstract

**Introduction:**

High‐resolution magnetic resonance imaging (MRI) of the cervical spinal cord is important to provide accurate diagnosis and pathological assessment of injuries. MEDIC (Multiple Echo Data Image Combination) sequences have been used in clinical MRI; however, a comparison of the performance of 2D and 3D MEDIC for cervical spinal cord imaging has not been reported. The aim of this study is to compare axial 2D and 3D MEDIC for the visualisation of the grey matter (GM) and white matter (WM) of the human cervical spinal cord.

**Methods:**

Eight healthy participants were scanned using Siemens Prisma^fit^ 3T MRI. T2*‐weighted gradient spoiled 2D and 3D MEDIC sequences were acquired at 0.4 × 0.4 × 3.0 and 0.3 × 0.3 × 3.0 mm resolutions, with the acquisition times of 6 and 7 min, respectively. Quantitative analyses of the images were made based on the image signal‐to‐noise ratio (SNR), contrast‐to‐noise ratio (CNR) and non‐uniformity (NU). Two independent radiologists (CS and FN), each provided Likert scoring assessments of anatomical visibility of the GM and WM structures and image clarity for all samples.

**Results:**

Quantitative evaluation showed that 3D MEDIC provided higher SNR, higher CNR and lower NU than 2D MEDIC. However, 2D MEDIC provided better anatomical visibility for the GM, WM and CSF, and higher image clarity (lower artefacts) compared to 3D MEDIC.

**Conclusions:**

2D MEDIC provides better information for depicting the internal structures of the cervical spinal cord compared to 3D MEDIC.

## Graphical abstract

2D MEDIC provided better quality images for depicting the cervical spinal cord's white and grey matter structures with reduced artefacts and superior clarity compared to the 3D MEDIC. This paper showed that the 2D MEDIC can potentially be used to obtain reliable measurements of atrophy and lesions in the internal spinal cord structures, which is important for diagnosis in diseases such as multiple sclerosis and spinal cord injury.

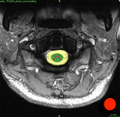

## Introduction

MRI has been widely used as a non‐invasive tool to assess cervical spine pathology as it demonstrates high resolution of the bone structure and soft tissue in multiple planes.^1^ The standard MRI sequences employed to perform such tasks are two‐dimensional (2D) T1‐weighted and T2‐weighed Fast Spin Echo, and T2* Gradient Echo (GRE).^2^ New sequences have been developed to provide improved T2‐weighted contrast, such as 2D Multiple Echo Recombined Gradient Echo (MERGE), and three‐dimensional (3D) spin echo sequences such as CUBE, VISTA and SPACE.^3^ 3D techniques offer benefits of high spatial resolution, high signal‐to‐noise ratio (SNR) and the absence of crosstalk between sections; however, they require long acquisition times and therefore they are often not suitable for clinical use.^4^

2D T1‐ and T2‐weighted fast spin echo (FSE) sequences at 3T have been shown to produce better visualisation of the cervical spinal cord than those at 1.5T.^2^ White et al. found that T2*‐weighted multi gradient echo (GRE) sequence produced good grey matter (GM) and white matter (WM) tissue contrast at 1.5T,^3^ but a detailed comparison of GRE sequences for cervical spinal cord imaging at 3T has not been reported. MR images acquired using 3T can have more pronounced artefacts than 1.5T, resulting from a combination of increased local magnetic field susceptibility and sensitivity to motion artifacts (respiratory movement and flow of the cerebrospinal fluid).^5^

New gradient technology (80mT/m gradients with >200mT/m/s slew rates), allows further development of spoiled T2* gradient combined echo sequences (known as MERGE, Multiple Echo Data Image Combination (MEDIC) or multi‐echo fast field echo (M‐FFE)) to achieve high image resolution and short acquisition times.^3^, ^6^ A small comparative study by Xiao et al, showed that 3D FFE could visualise the internal anatomy of the cervical spinal cord better than 2D FSE,^4^ but a 2D/3D comparison of the same sequences was not performed.

MEDIC refers to a T2*‐ spoiled gradient echo sequence consisting of a single radiofrequency excitation followed by multiple bipolar gradient echoes that are combined to form an image.^7^ The shorter echoes are used to increase SNR and the longer echoes to improve T2* weighted image contrast.^8^, ^9^ This sequence can be performed as a 2D or 3D acquisition and has been shown to provide good contrast between the GM and WM in the cervical spine in MRI 1.5T with a scan time of approximately 5 min.^6^, ^10^

This study compared the performance of 2D and 3D MEDIC sequences acquired with comparable scan times at 3T to obtain the best image quality for delineating the human cervical spinal cord structures. Our hypothesis was that 3D MEDIC could provide higher image resolution and quality for the delineation of the grey and white matter structures compared to 2D MEDIC. The MRI protocol included the anterior and posterior aspects of spinal roots and evaluated the GM and WM tissue contrast, SNR, image artefacts and anatomical structure visibility. A further aim of this study was to facilitate improvements in the quality of cervical spinal cord imaging for a wide range of clinical diagnoses.^10^, ^11^

## Methods

Eight healthy volunteers (four males, four females) were recruited and underwent MRI cervical spine scans on a 3T Prisma^fit^ (Siemens, Erlangen, Germany) at the Centre for Advanced Imaging. A 64‐channel head‐and‐neck coil was used for imaging. The participants had a mean age of 30.25 ± 6.52 years (mean ± SD). Each participant gave written consent, and the study was approved by The University of Queensland Human Ethics Committee. All data was de‐identified on the scanner.

### MRI protocol

The scan protocol consisted of a sagittal T2‐weighted FSE localiser, an axial 2D MEDIC and an axial 3D MEDIC. The MRI sequence parameters are shown in Table 1.

**Table 1:** 2D and 3D MEDIC sequence parameters.

	TR/TE (ms)	FA/ETL/NEX	FOV (mm)	Matrix	Voxel (mm)	Slice package	Phase over‐sampling	Scan time
2D FSE Localiser	4090/105	90/17/1	220 × 220	368 × 288	0.6 × 0.6 × 3.0	N/A	N/A	1 min 8 s
2D MEDIC	1000/14	30/4/2	180 × 135	384 × 230×21 slices	0.4 × 0.4 × 3.0	7 with 3 slices/ package	100%	6 min 11 s
3D MEDIC	38/17	11/3/2	133 × 192	400 × 576×30 slices	0.3 × 0.3 × 3.0	1	30%	7 min 20 s

2D = two‐dimensional, 3D = three‐dimensional, ETL = echo train length, FA = flip angle, FOV = field of view, FSE = fast spin echo, MEDIC = Multiple Echo Data Image Combination, NEX = number of excitations, TE = echo time, TR = repetition time.

The axial 2D MEDIC used a multi slice package mode, where each slice package was positioned in the centre of each cervical level. The first slice was positioned on the superior surface of the body of the vertebra (C1 to C7), and the group slices were set perpendicular to the spinal cord in the anterior/posterior (A/P) and left/right (L/R) directions. An anterior saturation band was used to diminish swallowing movement artefacts. The axial 3D MEDIC parameters were optimised from a study of cervical spondylotic myelopathy.^12^ The planning for 2D and 3D MEDIC is shown in the Supporting Information.

The 2D and 3D MEDIC parameters were chosen to obtain the best image quality whilst maintaining similar in‐plane resolutions and acquisition times. The 2D and 3D MEDIC had a similar T2* contrast based on the range of the TEs used. Minimum TR was selected for the number of slices. Compared to the 3D MEDIC, the 2D MEDIC required a longer TR to accommodate the number of slices. An Ernst angle calculation indicated that the 3D MEDIC would have a stronger T1‐weighting than the 2D MEDIC.

### Image processing

Axial images C1‐C7 were compared side by side between the 2D and the 3D MEDIC. To ensure that the spinal cord visualisation was done at the same level from C1 to C7, RadiAnt Digital Imaging Communication in Medicine (DICOM) viewer Software (Medixant, radiantviewer.com) was utilised to view the positions 2D and 3D MEDIC axial slices using the sagittal image localiser as a reference.

**Figure 1:**
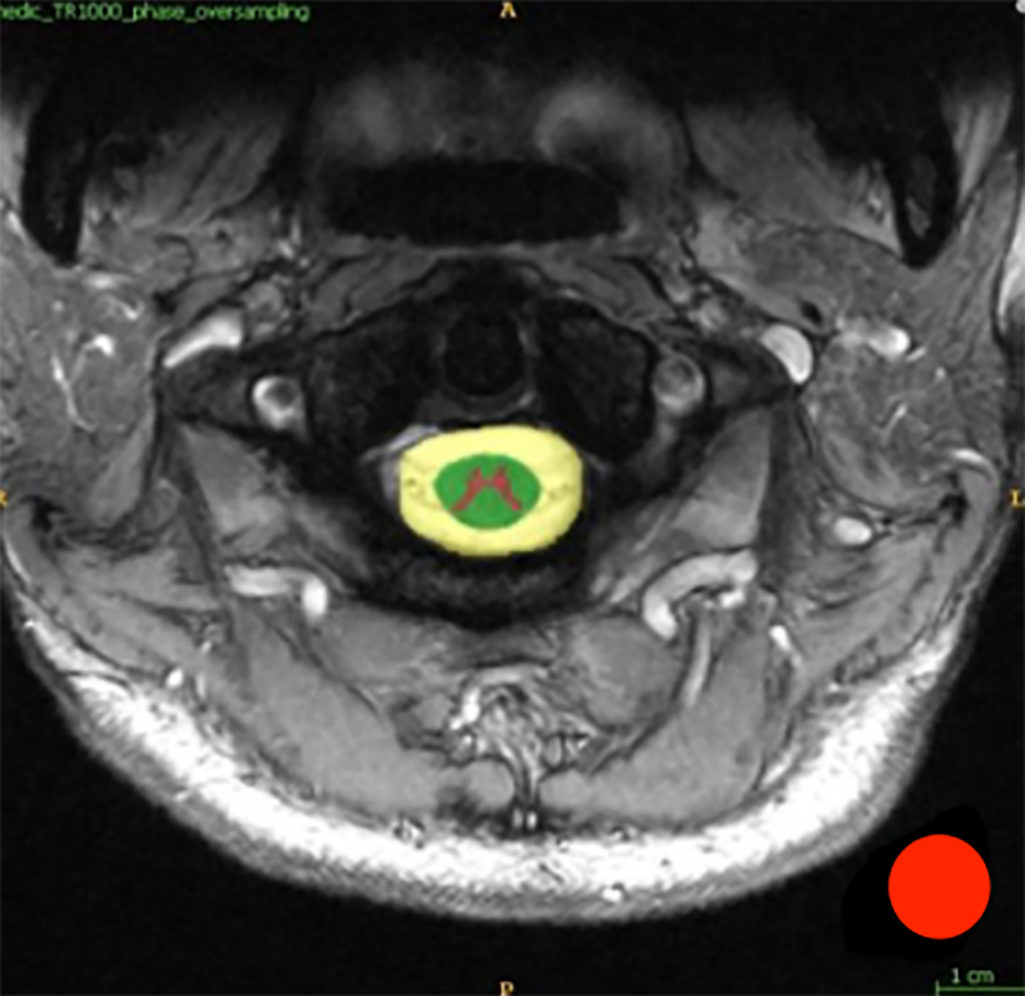
Regions of interests for the grey matter (purple), the white matter (green), the cerebrospinal fluid (yellow) and the background (red).

Regions of interest (ROI) measurement was performed using ITK‐SNAP (itksnap.org) to calculate the mean and standard deviation (SD) of signal intensities in the GM, WM, CSF and background noise of each cervical level of each participant. The polygon inspector and the brush tools were used to manually segment each anatomical ROI. The size of the ROIs (mm^2^) for the GM, WM and CSF were the same for the equivalent cervical level in each patient. They were anatomically constrained to patients cord structure and size. The entire anatomical region is used for the calculation of the SNR and CNR. For the measurement of the background signal, circular ROIs with 20 mm diameter were placed in the area outside the image, away from ghosting artefacts (Fig. 1). The signals within each ROI were calculated as an average of all voxel intensity within. This protocol was performed to provide more accurate and detailed calculation compared to previous studies, which used small circles as ROIs placed in the spinal cord structures.^4^, ^10^

Quantitative image assessment included signal‐to‐noise ratio (SNR), contrast‐to‐noise ratio (CNR) and non‐uniformity (NU).^13^

SNR measures the differences in signal intensities between the tissue of interest and the background noise. The ROIs used to measure the background noise were placed in similar location in each dataset to avoid any phase or ghosting artefacts. Higher SNR is desired as it will diminish the grainy appearance of the image.^7^ SNR was calculated as:

$SNRtissue_{\left(x\right)}=\frac{meanSItissue_{\left(x\right)}‐meanSIbackground_{\left(\mathrm{air}\right)}}{Standarddeviationofbackground_{\left(\mathrm{air}\right)}}$ where, x = a given tissue, SI = Signal Intensity.

CNR refers to the differences in signal intensities between two anatomical regions. An increase of CNR will improve the ability to delineate two different regions of interest.^14^ CNR was calculated as:^10^

$$CNRtissue_{\left(1,2\right)}=SNRtissue_{\left(1\right)}‐SNRtissue_{\left(2\right)}$$

NU refers to the presence of irrelevant intensity variation in the field of view.^15^ A low NU indicates an unblemished homogenous image with no/minimal noise and artefacts.^16^ NU was calculated as:

$$NU=\frac{\mathrm{StandardDeviationROI}}{\mathrm{MeanSignalintensityROI}}.$$

The mean signal intensity and the standard deviation of signal intensity were measured using the same ROI tissue area used in the CNR and SNR calculations.

Two experienced neuro‐radiologists (CS and FN) assisted this study as independent blinded observers to evaluate anatomical visibility and image clarity of all images of the cervical spinal cord.

Anatomical Visibility. Evaluation of the anatomical structures of the cervical spinal cord involved the identification of WM and GM structures, especially for the configuration of the H or butterfly shapes. For this assessment, a Likert scoring scale 1–5^17^ was used for image evaluation as follows: 1 = not visible, 2 = barely visible, 3 = adequately visible, 4 = good visibility, 5 = excellent visibility.

Image clarity. This includes detection of movement artefact, CSF pulsation or aliasing artefact that affected image quality. To evaluate the presence of artefacts on the images, a‐three‐point scoring scale was used as follows: 1 = extensive artefacts, therefore cannot be used for clinical diagnosis; 2 = moderate artefacts, can be used partially for diagnosis; 3 = minimum or no artefact, excellent use for diagnosis.

### Statistical analysis

Mean and standard deviations were calculated within each cervical level, across all participants from the 2D and 3D MEDIC datasets. Each cervical slice was treated as paired variables during the analyses. Non‐parametric, paired sample t‐tests were employed to evaluate differences in SNR, CNR, NU, anatomical visibility of GM, WM, CSF structures and image clarity. Statistical analyses were performed using Microsoft Excel. A *P*‐value < 0.05 was considered statistically significant.

Correlation analyses of image quality assessments. Two‐tailed Pearson’s correlation tests (R) were used to measure the correlations of 2D and 3D MEDIC image parameters (CNR of the GM/WM, and the SNR of the GM and WM) to the visibility of the GM structure. The statistical tests were performed using SPSS, and normality tests were used to confirm the type of data distribution.

## Results

### Image portfolio

**Figure 2:**
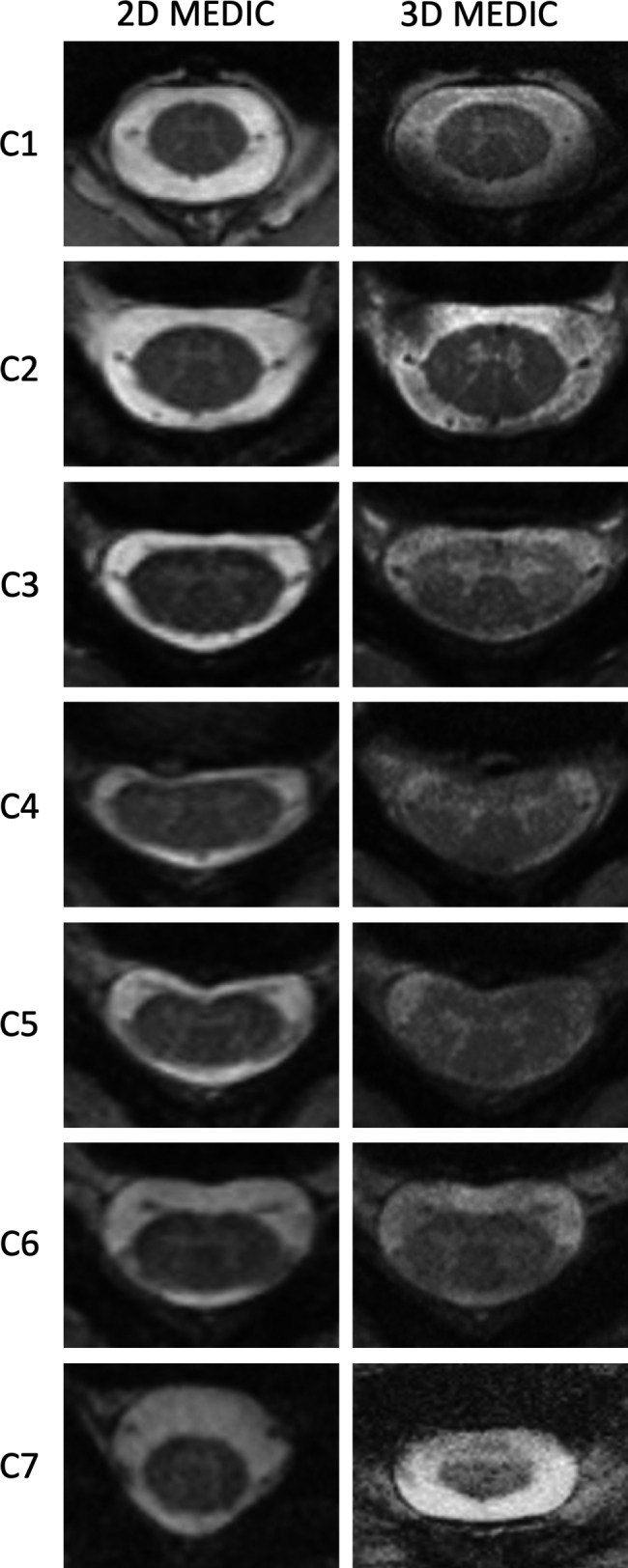
Imaging portfolio of one participant showing representative 2D and 3D MEDIC images. 2D = two‐dimensional, 3D = three‐dimensional, MEDIC = Multiple Echo Data Image Combination.

Cervical spine MRI of both axial 2D and axially resliced 3D images were arranged for each participant and the cervical spine levels. An example of participants’ cervical spine image portfolio can be seen on Figure 2. Image portfolio is available in the Supporting Information.

### SNR of cervical GM and WM tissues

**Figure 3:**
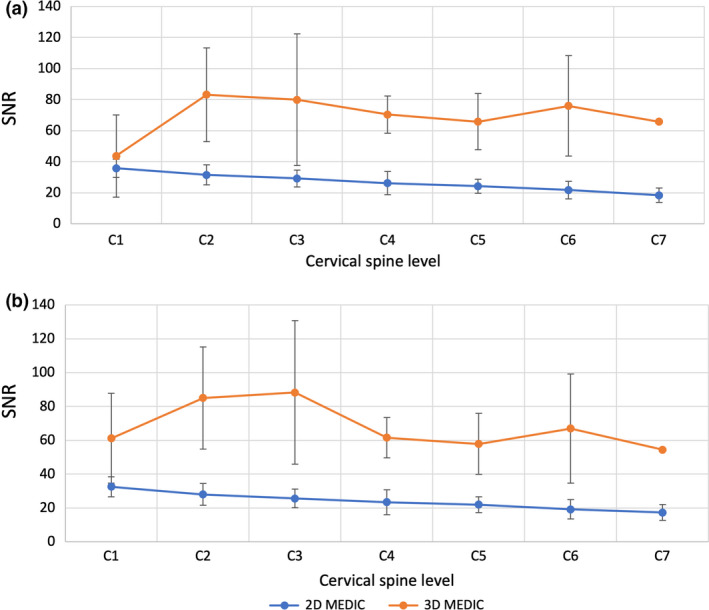
SNR comparison of 2D and 3D MEDIC images of the grey matter (A) and white matter (B) of the cervical spinal cord segments. Data are represented as mean ± standard deviation, *indicates statistically significant difference. 2D = two‐dimensional, 3D = three‐dimensional, MEDIC = Multiple Echo Data Image Combination.

There is no difference for the noise level in the WM for the 2D MEDIC (29.77 ± 4.70) and 3D MEDIC (27.39 ± 13.24, *P* = 0.50), but their average signal intensities are significantly different: 211.77 ± 49.61 and 450.02 ± 123.20 for the 2D and 3D MEDIC respectively (*P* < 0.01). The averaged GM SNR of the 3D MEDIC (69.23 ± 13.10) was higher than that of the 2D MEDIC (26.75 ± 5.93) (*p* < 0.01). A similar SNR was observed at C1, but the SNR of the 2D were significantly lower at the lower levels of the spine (Fig. 3A). 3D MEDIC also showed higher variability of SNR standard deviations when compared with 2D along the cervical spine, indicating better consistency the GM SNR levels in the 2D than the 3D. A similar averaged SNR profile was also observed for the WM (3D MEDIC: 67.87 ± 13.39, 2D MEDIC 23.99 ± 5.22, *P* < 0.01, Fig. 3B). Overall, the SNR values of the GM were higher than those of the WM in both 2D and 3D images (Table 2). This may be due to the fact that the GM contains fewer bound water molecules compared with the WM, and higher WM signal loss under T2*‐weighted sequences.^18^

**Table 2:** SNR and CNR comparison between 2D and 3D MEDIC.

	2D MEDIC	3D MEDIC	*P*‐value	t‐stat
GM SNR	26.75 ± 5.93	69.23 ± 13.10	0.0004	−7.09
WM SNR	23.99 ± 5.22	67.87 ± 13.39	0.0001	−9.41
GM/WM CNR	2.83 ± 0.74	8.86 ± 1.96	0.0004	−6.94

Data are represented as mean ± standard deviation, with the critical t‐value = 2.45. 2D = two‐dimensional, 3D = three‐dimensional, MEDIC = Multiple Echo Data Image Combination, GM = grey matter, WM = white matter, CNR = contrast‐to‐noise ratio, SNR = signal‐to‐noise ratio.

### CNR of spinal cord GM/WM structures

**Figure 4:**
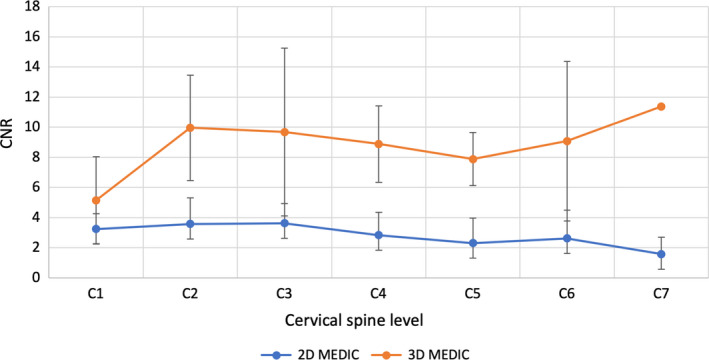
Grey and white matter CNR of the 2D and 3D MEDIC images along the cervical spinal cord segments. Data are represented as mean ± standard deviation, *indicates statistically significant difference. 2D = two‐dimensional, 3D = three‐dimensional, MEDIC = Multiple Echo Data Image Combination, CNR = contrast‐to‐noise ratio.

3D MEDIC showed a higher averaged CNR (8.86 ± 1.96) compared to 2D MEDIC (2.83 ± 0.74, *P* < 0.01). The CNR profile along the length of the cord parallels that of the SNR. A similar CNR was observed at C1 and the largest difference was found at C7 (Fig. 4). 3D MEDIC also showed higher variability of standard deviations and fluctuations in the CNR compared to the 2D. These observations indicated that although 3D MEDIC produced better GM/WM tissue contrast than 2D MEDIC, the later produced more consistent contrast throughout the cervical levels.

### Non‐Uniformity

The NU values of 2D and 3D MEDIC images were different for the white matter (*P* < 0.01) and the CSF (*P* < 0.01), but not for the grey matter (*P* = 0.72). Overall, 3D MEDIC showed a lower NU value (better image homogeneity) compared to the 2D MEDIC (Table 3).

**Table 3:** Non‐Uniformity comparison between 2D and 3D MEDIC.

	2D MEDIC	3D MEDIC	*P*‐value	t‐stat
GM	9.55 ± 0.92	9.86 ± 0.92	0.7190	−0.38
WM	18.23 ± 0.61	12.06 ± 1.18	0.0016	5.41
CSF	23.60 ± 0.75	20.72 ± 2.50	0.0091	3.79

Data are represented as mean ± standard deviation, with the critical t‐value = 2.45. 2D = two‐dimensional, 3D = three‐dimensional, CSF = cerebrospinal fluid, MEDIC = Multiple Echo Data Image Combination, GM = grey matter, WM = white matter.

### Anatomical visibility of cervical spinal cord structures

**Figure 5:**
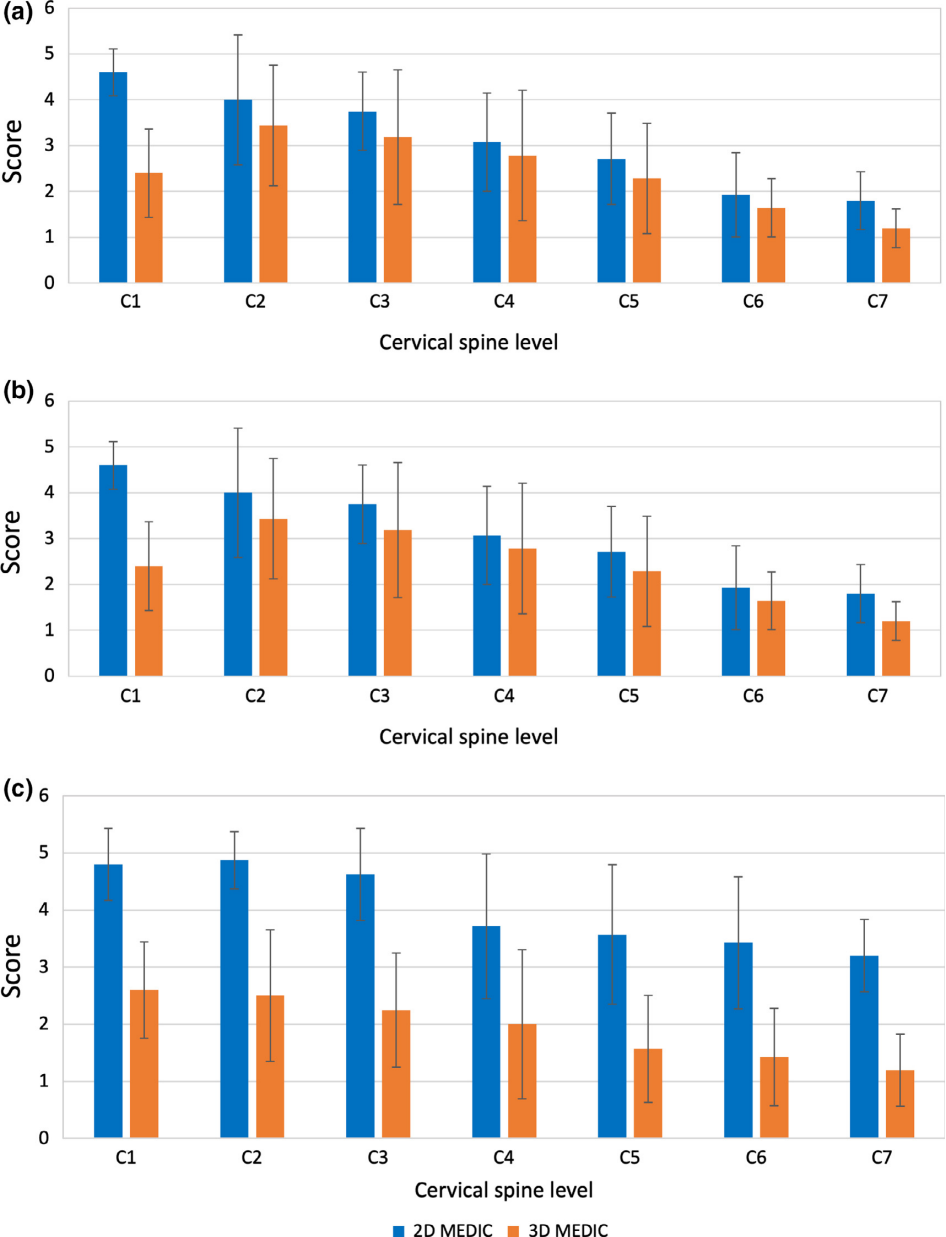
The anatomical structure visibility scores of the grey matter (A), white matter (B) and cerebrospinal fluid (C), of the 2D and the 3D MEDIC images. Data are represented as mean ± standard deviation, * indicates statistically significant difference. 2D = two‐dimensional, 3D = three‐dimensional, MEDIC = Multiple Echo Data Image Combination.

The visibility scores of the grey and white matter structures from C2 to C7 were similar (Fig. 5A and B), a significant difference was observed only at C1 (*P* < 0.01). From C2 to C7, the visibility of the grey matter for 3D and 2D decreased steadily across the C2 to C7. A pairwise statistical t‐test performed for the whole cervical levels showed that there was a significant difference between 3D and 2D MEDIC for grey and white matter visibility (*P* = 0.03). 2D MEDIC showed a higher CSF visibility score compared to the 3D (Fig. 5C). The CSF visibility score at the C1 level is 2.6 ± 0.84 for 3D MEDIC and 4.8 ± 0.63 for 2D MEDIC. These values were relatively stable from C1 to C3 before it declined at level C4 and decreased steadily towards level C7. Pairwise statistical t‐test of the whole cervical levels showed that there was a significant difference between 3D and 2D MEDIC for the visibility of CSF (*P* < 0.01). The visibility and statistical test results are shown in Table 4.

**Table 4:** A comparison between 2D and 3D MEDIC image visibility.

	2D MEDIC	3D MEDIC	*P*‐value	t‐stat
GM	3.12 ± 1.06	2.42 ± 0.80	0.033	2.77
WM	3.12 ± 1.06	2.42 ± 0.80	0.033	2.77
CSF	4.03 ± 0.71	1.94 ± 0.55	<0.001	23.29

Data are represented as mean ± standard deviation, with the critical t‐value = 2.45. 2D = two‐dimensional, 3D = three‐dimensional, CSF = cerebrospinal fluid, MEDIC = Multiple Echo Data Image Combination, GM = grey matter, WM = white matter.

### Image clarity

**Figure 6:**
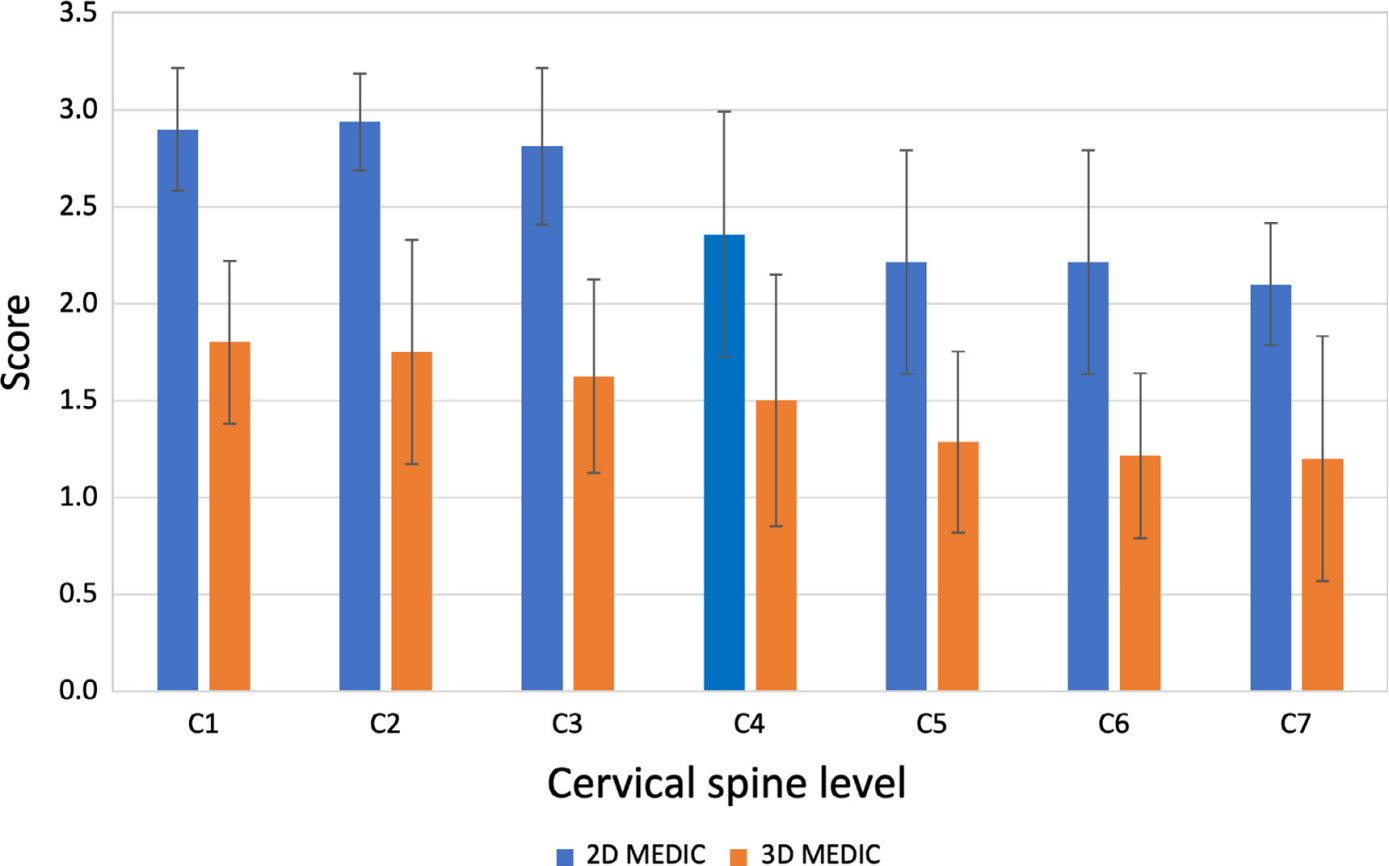
The image clarity scores of the 2D and the 3D MEDIC images. Data are represented as mean ± standard deviation, *indicates statistically significant difference. 2D = two‐dimensional, 3D = three‐dimensional, MEDIC = Multiple Echo Data Image Combination.

The image clarity scores of the 2D and 3D MEDIC are shown in Figure 6. 2D MEDIC has significantly higher image clarity score (2.51 ± 0.36) compared to 3D MEDIC (1.48 ± 0.25) (*P* < 0.01). The graph indicates that 3D MEDIC produces more artefacts than 2D MEDIC across all slices. The type of artefact seen on the images was motion artefact.

### Correlations between image quality parameters

Positive and strong correlations were found between the visibility of the GM in 2D MEDIC vs GM/WM CNR (*R* = 0.834, *P* = 0.02), vs GM SNR (*R* = 0.982, *P* < 0.01) and vs WM SNR (*R* = 0.990, *P* < 0.01). A strong correlation was also observed between the visibility of GM and 2D image quality (*R* = 0.941, *P* < 0.01). For 3D MEDIC, such correlations were not statistically significant. A medium strength correlation was found between the visibility of GM and the image quality (*R* = 0.792, *P* = 0.03). These figures are summarised in Table 5.

**Table 5:** Correlations between quantitative and qualitative image parameters.

Parameter comparison	Pearson’s *R* Correlation	*P*‐value
*2D MEDIC*		
Visibility of GM vs. CNR of GM/WM	0.834	0.020
Visibility of GM vs. SNR of GM	0.982	<0.001
Visibility of GM vs. SNR of WM	0.990	<0.001
Visibility of GM vs. Image clarity	0.941	0.002^*^
*3D MEDIC*		
Visibility of GM vs. CNR of GM/WM	−0.134	0.775
Visibility of GM vs. SNR of GM	0.684	0.134
Visibility of GM vs. SNR of WM	0.782	0.066
Visibility of GM vs. Image clarity	0.792	0.034

2D = two‐dimensional, 3D = three‐dimensional, MEDIC = Multiple Echo Data Image Combination, GM = grey matter, WM = white matter, CNR = contrast‐to‐noise ratio, SNR = signal‐to‐noise ratio.

## Discussion

This study has compared axial MR images acquired using 2D and 3D T2*‐weighted MEDIC sequences for the visualisation of the grey and white matter of the human cervical spinal cord at 3T. Theoretically, the 3D acquisition should provide a number of benefits over the 2D, for example visualising the whole cervical spinal column in one acquisition and higher spatial resolution. This study also investigated each sequence’s susceptibility to physiological artefacts over a medium length (six to seven mins) of acquisition time.

The characterisation of 2D/3D MEDIC sequences is important for selecting the correct protocol for measuring the internal cord structures enabling accurate diagnosis, prognosis and monitoring of therapy.^19^ The ability to detect abnormal changes in the spinal cord anatomical structures are pivotal in determining topographic involvement in neurological diseases, to gain insights of pathophysiology and to understand the location and the extent of the disruption in specific cord areas due to injury or disorders in relation to clinical presentations.

2D and 3D MEDIC data showed that the SNR, CNR and visual anatomical structure clarity parameters decreased from the superior to inferior of cervical spinal cord (C1‐C7) levels. These global changes may be related to the decrease of the volume of the spinal canal and increased partial volume effect from the upper cervical segments towards C7‐T1.^20^ The SNR and CNR of 3D MEDIC images were found higher than that of the 2D images. Tissue CNR is directly dependent on the SNR, and therefore, image SNR is an important parameter in achieving good WM/GM tissue contrast. Higher SNR in the 3D MEDIC images was obtained despite the acquisition using a much shorter TR than the 2D sequence, suggesting that the gain in SNR was more dependent upon the number of slice phase encoding steps in the 3D acquisition.^7^

It is important to note that image quality depends not only on high SNR and CNR but also on the visibility of the regions of interest and the absence of image artefacts. Overall, the 3D MEDIC has a lower image quality, a lower Non‐Uniformity and higher variabilities in the SNR and CNR levels (higher standard deviation) compared to the 2D MEDIC. The quantitative SNR and CNR parameters both showed strong correlations with the visibility of the GM structure in the 2D MEDIC, but not in the 3D MEDIC. There were strong correlations between the image quality (the absence of image artefacts) and the visibility of the GM structures in both 3D and 3D MEDIC. This data indicates that although 3D MEDIC can produce high SNR and CNR, it could not sufficiently provide clear visualisation of the spinal cord GM structures.

3D MEDIC images contained more motion artefacts than the 2D. The physiological movement in the spinal cord imaging is typically more problematic compared to brain imaging. The cord’s proximity to the lungs, throat and oesophagus is the major contributing factor. Further, motion artefacts are exacerbated in 3D datasets due to sensitivity to phase encoding errors, in addition to a typically longer acquisition time compared with 2D.^14^ Therefore, for clinical practice, it will be preferable to use 2D MEDIC instead of 3D MEDIC for trauma patients in order to hasten the acquisition and provide more reliable results.

Further improvements of the 3D MEDIC are required to reduce artefacts and scan time. These may include obtaining a smaller slab for specific levels of cervical imaging and applying a gating image technique (however, both would increase the scan time); using non‐Cartesian k‐space ordering, spiral or blade; or using a parallel imaging with a neck coil.^7^, ^21^

## Conclusion

2D MEDIC produced superior images for visualising the internal architecture of the cervical spinal cord compared to 3D MEDIC, as the latter was more prone to movement artefacts. Further development of 3D MEDIC is required before it can be used for routine clinical imaging in the cervical spinal cord.

## Conflict of Interest

The authors declare no conflict of interest.

## Supporting information

Supporting Information: Planning for 2D and 3D MEDIC; Image Portfolio.
