# Supplementary material for: Comparison between 2D and 3D MEDIC for human cervical spinal cord MRI at 3T
Source: J Med Radiat Sci. 2020 Sep 15;68(1):4–12. doi: 10.1002/jmrs.433 (PMC7890925; doi:10.1002/jmrs.433)
Supplement: Supplementary file 1 — Supporting Information: Planning for 2D and 3D MEDIC; Image Portfolio. [file JMRS-68-4-s001.pdf]

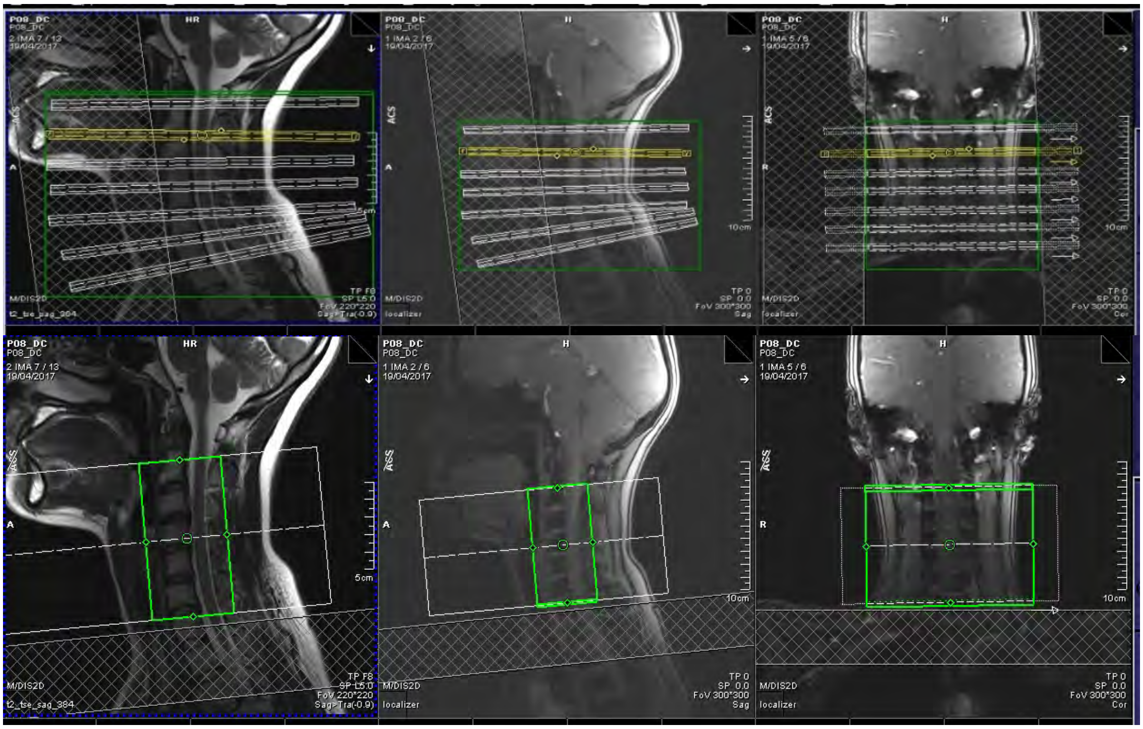

Planning for 2D and 3D MEDIC. (Top) seven 2D slice packages were placed in the centre of each cervical spinal cord. (Bottom) a single slab used to for 3D MEDIC. 2D = two-dimensional, 3D = three-dimensional, MEDIC = Multiple Echo Data Image Combination

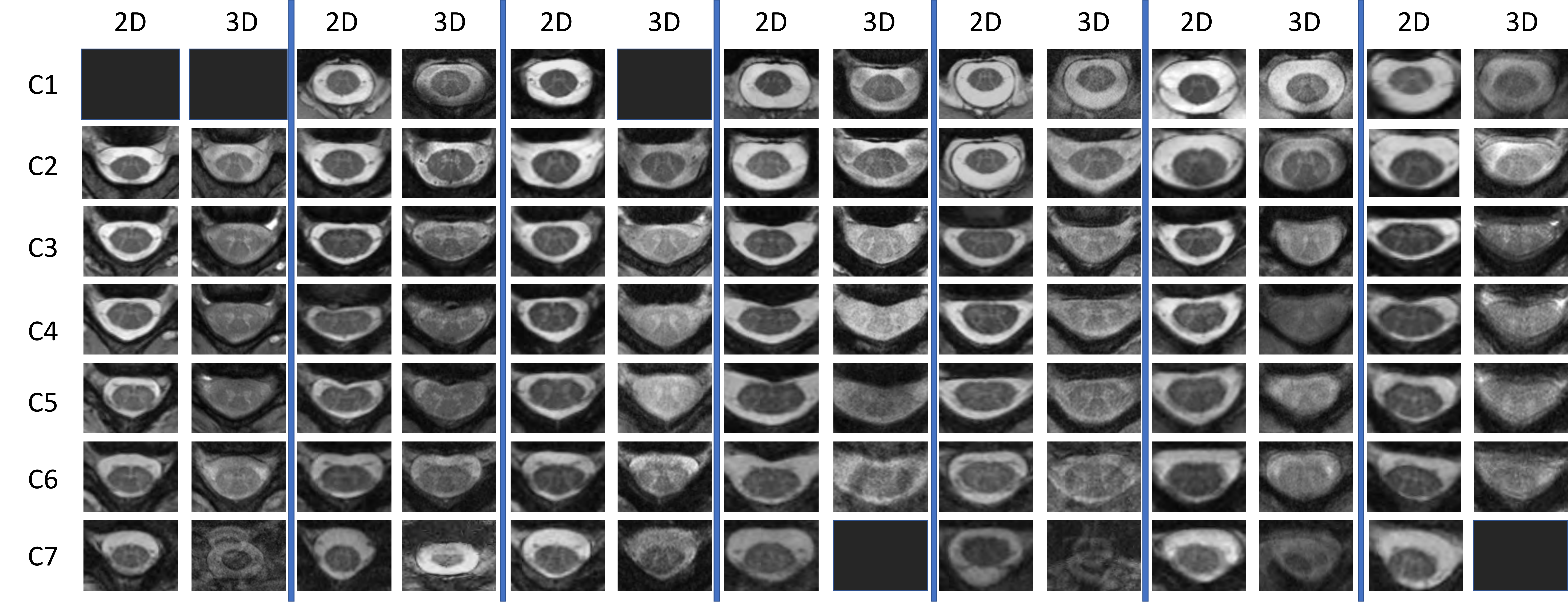

Cervical spinal cord two-dimensional (2D) and three-dimensional (3D) Multiple Echo Data Image Combination image portfolio
